# The complete chloroplast genome of *Clematis florida* Thunb. (Ranunculaceae), an ornamental and medicinal plant from Henan province, China

**DOI:** 10.1080/23802359.2022.2049460

**Published:** 2022-03-09

**Authors:** Yan Dong, Qingsong Zhu, Jianhua Yue

**Affiliations:** Xinyang Agriculture and Forestry University, Xinyang, Henan, People’s Republic of China

**Keywords:** *Clematis florida* Thunb., chloroplast genome, phylogeny, Ranunculaceae

## Abstract

*Clematis florida* Thunb. is a herbaceous and perennial plant native to East Asia. The plant is resistant to cold but sensitive to heat. It is an ornamental and medicinal plant that has great commercial potential. Here, we assembled and characterized the complete chloroplast (cp) genome of *C*. *florida*. The cp genome of *C*. *florida* was characterized by Illumina pair-end sequencing and is 159,606 bp in total length. The genome includes a large single-copy (LSC) region of 79,467 bp, a small single-copy (SSC) region of 18,057 bp, and a pair of inverted repeats (IR) regions of 31,041 bp. The genome contains 135 genes including 91 protein-coding, 36 tRNA, and eight rRNA genes. Phylogenetic analysis based on 18 *Clematis* species indicates that *C*. *florida* is closely related to *C. fusca* in the Ranunculaceae. The phylogenetic relationships and taxonomic status of *C*. *florida* revealed by cp genome were consistent with the previous molecular studies, and can serve as a reference for future studies on molecular biology, evolution, and taxonomy in the genus *Clematis*.

The genus *Clematis* contains about 355 species, many of which are used to study plant modeling, flower form, colors and florescence (Wang et al. 2021). One of these species, *Clematis florida* Thunb. (First mentioned in 1784, see in http://www.iplant.cn/) is a herbaceous, perennial plant native to East Asia (Sheng et al. 2014). This species is resistant to cold temperature but is heat sensitive (Jiang et al. 2020). This species is well-known for its high ornamental value (Jiang et al. 2020). *C. florida*, also called ‘Queen of vines’, is commonly used for landscaping and floriculture, which is a popular climbing plant worldwide (Jiang et al. 2020). It is also a plant source of many medicinal active ingredients including antioxidant and anti-inflammatory metabolites (Jung et al. 2021, Wang et al. 2021). The cp genome has a maternal inheritance and conserved structure, and has been used to examine the developmental and phylogenetic relationships of plants (Wang et al. 2018). To better understand the phylogenetic position of *C. florida*, we assembled and analyzed the complete cp genome of *C*. *florida* using Illumina pair-end sequencing data.

*Clematis florida* leaves were collected from Xinyang, Henan Province, China (Xinyang Agriculture and Forestry University: 114°13′E, 32°17′N) and preserved in liquid nitrogen. Later these specimens (Bio-sample accession: SAMN20060056) were stored at −80 °C at the Horticultural Plant Biotechnology Laboratory, Xinyang Agriculture and Forestry University. A specimen was deposited at the Herbarium of the Horticultural Plant Biotechnology Laboratory, Xinyang Agriculture and Forestry University (Contact person: Jianhua Yue, jhyues@163.com) under the voucher code CF5601. Genomic DNA was extracted by the CTAB method (Odahara et al. 2009). After the DNA extraction from leaf tissues, its quantification was validated using NanoDrop spectrophotometer 2000. The library construction and sequencing were performed on the Illumina High-throughput sequencing platform (NovaSeq 6000). Approximately 11.9 GB of raw data was generated with 150 bp paired-end read lengths. The data were filtered using NOVOPlasty (Dierckxsens et al. 2016). The complete plastid genome of *C. fusca* (GenBank accession: KM652489) was chosen as the reference. The plastid genome was assembled by GetOrganelle, then viewed and edited by Bandage (Wick et al. 2015). The cp genome annotation was performed by Geneious v 11.1.5 (Biomatters Ltd, Auckland, New Zealand) (Kearse et al. 2012).

The complete cp genome of *C*. *florida* is circular and 159,606 bp in length, with 37.51% GC content. The total plastid genome consisted of four distinct regions including the LSC region of 79,467 bp, SSC region of 18,057 bp, and a pair of IR regions of 31,041 bp. The complete cp genome consists of 135 genes, including 91 protein coding, 36 tRNA, and eight rRNA genes. The cp genome structure of *C*. *florida* was very close to *C. fusca*, which showed a cp genome of 159,609 bp in length, the LSC of 79,478 bp, SSC of 18,053 bp, and a pair of IR regions of 31,039 bp (Park and Park 2016).

To explore the evolutionary relationship of *C*. *florida* to other species in the genus, 18 *Clematis* sequences were selected and *Agapanthus praecox* served as the outgroup for the phylogenetic analysis. All of the genomes were downloaded from NCBI GenBank. The sequences were aligned by MAFFT v7.307 using routine settings (Katoh and Standley 2013), and the phylogenetic tree was constructed by MEGA X (Kumar et al. 2018). The robustness of the topology was estimated using 1000 bootstrap replicates with the maximum likelihood method and nucleotide substitution model Tamura-Nei following Nguyen et al. (2015). The phylogenetic tree revealed that *C*. *florida* was fully resolved in a clade with *C. fusca* (Figure 1). The result was highly consistent with the phylogenetic relationships and taxonomic status of the *Clematis* species by using nuclear ITS and plastid atpB-rbcL fragments methods (Zhang et al. 2015). The analysis of the cp genome of *C*. *florida* provides excellent genetic information for further studies of this species and the taxonomy, phylogenetics, and evolution of the Ranunculaceae.

**Figure 1.**
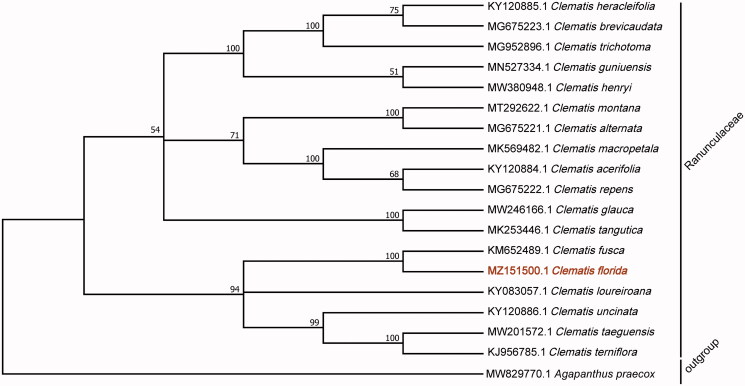
Phylogenetic analysis based on the complete cp genomes. The bootstrap values were shown on the nodes, the species and GenBank accession number were shown at the end of each branch.

## Data Availability

The data that support the findings of this study are openly available at https://www.ncbi.nlm.nih.gov/. The complete cp genome has been deposited in GenBank with accession number MZ151500. And the associated Bio-project, SRA, Bio-sample numbers are PRJNA743757, SRR15041511, and SAMN20060056 respectively.
